# Inflammation and Immune Response of Intra-Articular Serotype 2 Adeno-Associated Virus or Adenovirus Vectors in a Large Animal Model

**DOI:** 10.1155/2012/735472

**Published:** 2012-01-11

**Authors:** Akikazu Ishihara, Jeffrey S. Bartlett, Alicia L. Bertone

**Affiliations:** ^1^Comparative Orthopedic Research Laboratories, Department of Veterinary Clinical Sciences, The Ohio State University, 1900 Coffey Road, Columbus, OH 43210, USA; ^2^Gene Therapy Center, The Research Institute at Nationwide Children's Hospital, Columbus, OH 43205, USA; ^3^Department of Pediatrics, The Ohio State University, Columbus, OH 43205, USA

## Abstract

Intra-articular gene therapy has potential for the treatment of osteoarthritis and rheumatoid arthritis. To quantify in vitro relative gene transduction, equine chondrocytes and synovial cells were treated with adenovirus vectors (Ad), serotype 2 adeno-associated virus vectors (rAAV2), or self-complementary (sc) AAV2 vectors carrying green fluorescent protein (GFP). Using 6 horses, bilateral metacarpophalangeal joints were injected with Ad, rAAV2, or scAAV2 vectors carrying GFP genes to assess the in vivo joint inflammation and neutralizing antibody (NAb) titer in serum and joint fluid. In vitro, the greater transduction efficiency and sustained gene expression were achieved by scAAV2 compared to rAAV2 in equine chondrocytes and synovial cells. In vivo, AAV2 demonstrated less joint inflammation than Ad, but similar NAb titer. The scAAV2 vectors can induce superior gene transduction than rAAV2 in articular cells, and both rAAV2 and scAAV2 vectors were showed to be safer for intra-articular use than Ad vectors.

## 1. Introduction

In elderly people, joint disorders including osteoarthritis and rheumatoid arthritis remain major causes of mobility loss [1]. Gene therapy has a great potential for the treatment of these conditions by using intra-articular gene delivery, such as previously described for interleukin-1 receptor antagonist (IL-1Ra) [2, 3], transforming growth factor-*β*1 [4], or tumour necrosis factor alpha antagonist protein [5]. Direct intra-articular administration of gene delivery vectors is an attractive and effective strategy to transduce articular cartilage and synovium with therapeutic genes, because joints are discrete and accessible cavities that can be easily injected [6].

A choice of viral vectors has been used to introduce therapeutic genes to intra- and periarticular tissues including adenovirus (Ad) [7, 8] and serotype 2 adeno-associated virus (AAV2) [9–11], and Ad vectors have been shown to induce rapid and successful transgene expression, but intra-articular usage has been restricted due to robust inflammatory and immune reactions, as well as limited by transient transgene expression [6–8]. In contrast, recombinant AAV2 (rAAV2) has gained much attention as a safer vector for gene delivery to joints, because it can induce sustained transgene expression in the absence of significant inflammation [6, 9–11]. In addition, rAAV2-mediated joint therapy may be advantageous by its greater penetration into articular cartilage, likely due to smaller particle size [12]. Moreover, AAV2 vectors have been modified to package self-complementary DNA, capable of bypassing the rate-limiting step of second-strand synthesis in order to accelerate and improve the transduction efficiency [12, 13]. Such self-complementary AAV2 (scAAV2) vectors have been shown to effectively transduce chondrocytes and synovial cells [14] and cartilage explants [12] in vitro, and induce superior transgene expression in cartilage and synovium tissues after injection of 5 × 10^11^ particles in vivo in rodent models [3]. Taken together, intra-articular administration of scAAV2 vectors has potential for future clinical application, but the inflammation and immune reactions, cell tropism, and application to larger joints as in people have not been directly compared among rAAV2, scAAV2, or Ad vectors in vivo.

The objectives of our study were to assess the relative gene transduction and duration of expression among Ad, rAAV2, and scAAV2 vectors carrying green fluorescent protein (GFP) in chondrocytes and synovial cells in vitro and compare the inflammation and immune response by the intra-articular administration of Ad, rAAV2, and scAAV2 vectors in vivo in equine joints of similar size to human joints. We hypothesized that scAAV2 vectors would show accelerated and greater gene transduction in vitro compared to rAAV2 and induce less inflammation and immune response in vivo compared to Ad vectors.

## 2. Materials and Methods

### 2.1. Viral Vector Production

Recombinant E-1 deleted human serotype 5 adenovirus preparations, single-stranded human serotype 2 AAV2, and scAAV2 containing GFP under the control of the cytomegalovirus (CMV) promoter were generated and purified by cesium chloride density gradient method [12, 15]. Particle count of Ad vectors was determined by optical density at 260 nm, and particle titers of rAAV2 and scAAV2 vectors were determined by DNase-resistant particle (DRP) values using real-time PCR assay as described previously [16].

### 2.2. In Vitro Experimental Design and Vector Administration

Articular cartilage and synovium were harvested from the tarsocrural joints of 6 healthy adult horses immediately after euthanasia. Chondrocytes and synovial cells were isolated by type I collagenase tissue digestion for 8 hours and cultured in DMEM supplemented with L-glutamine (300 *μ*g/mL), penicillin (30 *μ*g/mL), streptomycin (30 *μ*g/mL), and 10% fetal bovine serum at 37°C in a 5% CO_2_ atmosphere [17]. After expanding for 3–5 passages, the equine chondrocytes and synovial cells were placed in 48-well plates at a density of 10,000 cells/well (day 1). Twenty-four hours later (day 0), the DMEM was aspirated, and the duplicate wells were treated with 200 *μ*L of Gey's balanced salt solution (GBSS) containing Ad-GFP at 1 × 10^2^, 1 × 10^3^, or 1 × 10^4^ particles/cell (1 × 10^6^, 1 × 10^7^, or 1 × 10^8^ particles/well, resp.), or rAAV2-GFP, or scAAV2-GFP at 1 × 10^4^, 1 × 10^5^, or 1 × 10^6^ DRP/cell (1 × 10^8^, 1 × 10^9^, or 1 × 10^10^ DRP/well, resp.). After 2-hour incubation at 37°C in a 5% CO_2_ atmosphere, the GBSS was aspirated and replaced with 1 mL DMEM. The chondrocytes and synovial cells were cultured for 6 weeks (42 days), while the DMEM were changed at days 7, 14, 17, 21, 24, 28, 30, 32, 35, 37, 39, and 42.

### 2.3. In Vitro Gene Transduction

Transduction of the Ad-GFP, rAAV2-GFP, or scAAV2-GFP-treated cells was determined by the ratio of GFP-expressing cells to the total number of cells observed via fluorescent microscopy within the 5 fields of 25 × 25-*μ*m area under 200x magnification [17]. The % transduction was assessed at days 2, 7, 10, 14, 17, 21, 24, 28, 31, 35, 38, and 42. The % transduction values from the duplicate culture wells were averaged.

### 2.4. In Vivo Experimental Design and Vector Administration

**Figure 1:**
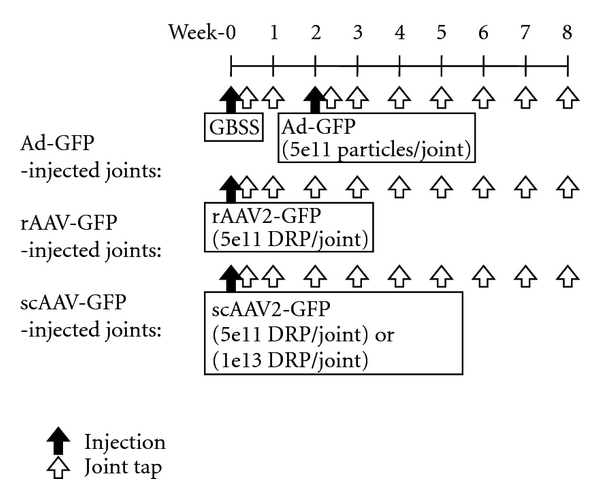
In vivo experimental design of intra-articular administration of adenoviral (Ad), recombinant aden-associated viral (rAAV2), or self-complementary AAV (scAAV2) vectors containing green fluorescent protein (GFP).

The in vivo experimental design is summarized in Figure 1. All procedures were approved by the Institutional Laboratory Animal Care and Use Committee at The Ohio State University.

 By using 4 healthy adult horses (3 Thoroughbreds and 1 Quarter horses) aged 5–12 years (median age: 7) and weight 418–536 kg (mean weight: 429 kg), 8 metacarpophalangeal joints (2 joints/horse) were treated by the intra-articular administration of GBSS, Ad-GFP (5 × 10^11^ particles/joint), rAAV2-GFP (5 × 10^11^ DRP/joint), scAAV2-GFP (5 × 10^11^ DRP/joint), or scGFP (1 × 10^13^ DRP/joint). In joints assigned randomly to be injected with Ad, GBSS was injected at day 0 and the Ad-GFP at day 14 after a normalization period, because Ad vectors were expected to induce rapid transgene expression. The GBSS-induced inflammation was assessed between day 0 and 14 prior to the Adinjection.

Intra-articular administration of viral vectors was performed while horses were standing and sedated with xylazine hydrochloride (i.v., 1 mg/kg). After the aseptic preparation of skin over the lateral aspect of the metacarpo/tarsophalangeal joints, the limb was held off the ground at approximately 120 degree flexion, and a 20 G 1.5 inch needle was inserted into the joint space between the proximal sesamoid bone and lateral distal condyle of the third metacarpal/tarsal bone by passing through the lateral collateral sesamoidean ligament. The intra-articular administrations of assigned treatments were performed only after the 2 mL joint fluid was collected to ensure the needle placement.

### 2.5. In Vivo Inflammatory and Immune Response

Physical examinations were performed weekly after the joint injection by evaluating circumference of the injected joints and pain-free range of joint motion. Circumference of the injected joint was recorded as the mean of 3 measurements obtained over the injection site by use of a cloth measuring tape [18]. Range of pain-free motion was recorded as the mean of 3 goniometer measurements of the flexed joint immediately before elicitation of an aversion response such as lifting of the head or movement of the limb forward/backward [18]. Lameness grades were assigned to each horse by an experienced equine clinician (ALB) using the American Association of Equine Practitioners' lameness with 0.5 increments [19], whereas a joint on the nonlame limb was assigned as 0 lameness grade.

Joint fluid samples were collected weekly by using the same technique described above and analyzed for cell count and total protein concentration. The interleukin 1 beta (IL-1*β*) and IL-1Ra protein concentration in the joint fluid was quantified by ELISA (Equine IL-1Ra DuoSet, R&D Systems, Minneapolis, MN). Serum samples were collected weekly, and neutralizing antibody (NAb) titer for Ad and AAV2-vectors were measured for serum and joint fluid samples from all joints. Titers were determined by analyzing the ability of serum antibody to inhibit Ad-GFP (1 × 10^4^ particles/cell) or rAAV2/scAAV2-GFP infection (1 × 10^6^ DRP/cell) on Human carcinoma cells (HeLa cells: 5 × 10^3^ cells/well in 96-well plates) at 48 hours [15]. By applying twofold dilution series of sample serum, the NAb titer was calculated as the highest serum dilution inhibiting Ad or rAAV2 or scAAV2-GFP transduction by >50%.

### 2.6. Statistical Analysis

Repeated-measure analysis of variance (ANOVA) (SAS Institute Inc., Cary, NC) was used to evaluate the effects of Ad or rAAV2 or scAAV2-mediated GFP gene delivery with the posttest multiple comparisons between the treatment groups at each time point using *Proc Mixed* statistical models for continuous outcomes and *Genmod* statistical models for categorical outcomes. Significance level was set at *P* < 0.05 for all analyses.

## 3. Results

### 3.1. In Vitro Gene Transduction

**Figure 2:**
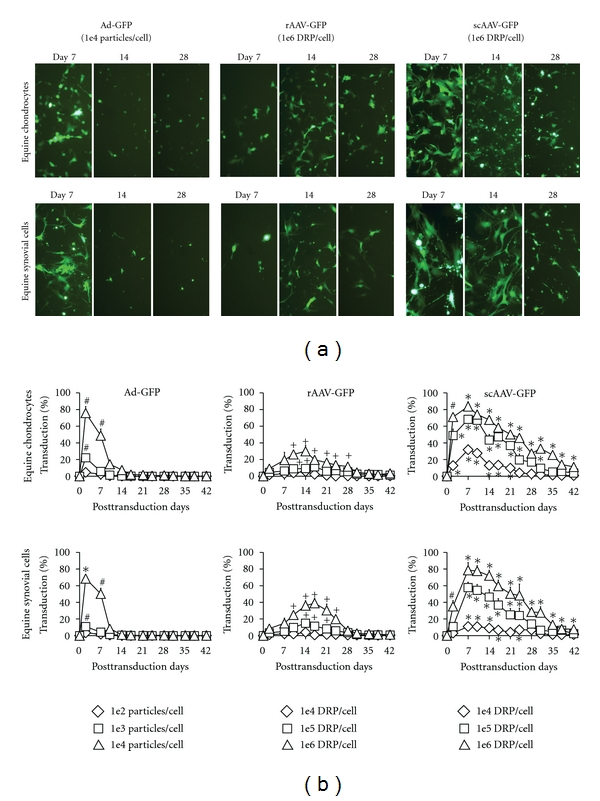
In vitro gene transduction efficiency in equine chondrocytes and synovial cells transfected by adenoviral (Ad), recombinant adeno-associated viral (rAAV2), or self-complementary AAV (scAAV2) vectors carrying green fluorescent protein (GFP) genes at three different dosages; low, medium, and high dosage levels for Ad-GFP were 1e4, 1e5, and 1e6 particles per cell, and low, medium, and high dosage levels for rAAV2/scAAV2 were 1e6, 1e7, and 1e8 DRP per cell. (a) Representative fluorescent microscopic images (approximately 90% cell confluence at day 7, and 100% cell confluence at day 14 and 28) and (b) the computed %transduction of equine chondrocytes and synovial cells. *Significantly greater gene transduction in same dosage level compared to Ad and rAAV2 vectors. ^+^Significantly greater gene transduction in same dosage level compared to Ad vectors. ^#^Significantly greater gene transduction in same dosage level compared to rAAV2 vectors.

Self-complementary AAV2 vectors produced greater and more rapid GFP gene transduction (Figures 2(a) and 2(b)) compared to rAAV2 vectors in equine chondrocytes and synovial cells. Also, scAAV2 vectors showed more sustained GFP gene transduction (Figures 2(a) and 2(b)) compared to Ad vectors when they showed comparable % transduction in equine chondrocytes and synovial cells, where the onset of GFP gene transduction (Figures 2(a) and 2(b)) was equally rapid to Ad vectors.

### 3.2. In Vivo Inflammatory and Immune Response

**Figure 3:**
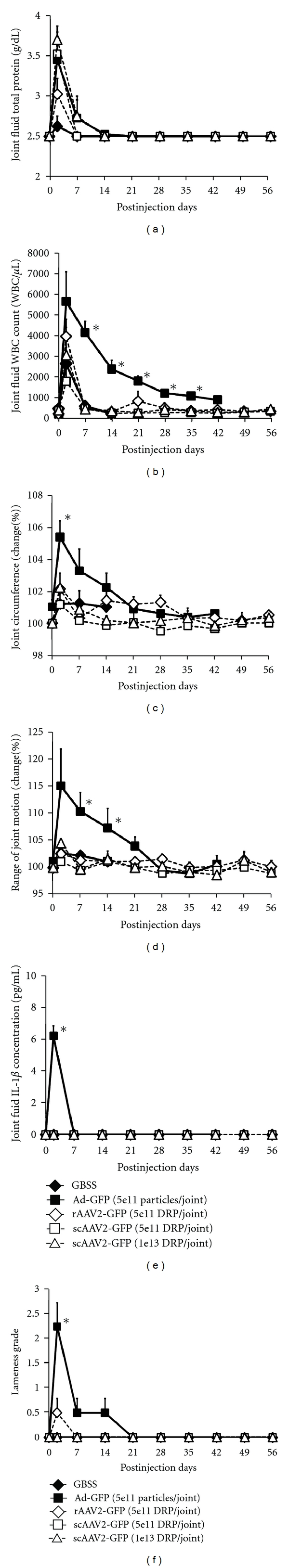
In vivo inflammatory response of equine metacarpal-phalangeal joints treated by the intra-articular administration of adenoviral (Ad), recombinant adeno-associated viral (rAAV2), or self-complementary AAV (scAAV2) vectors carrying green fluorescent protein (GFP) genes. (a) Joint fluid protein concentration, (b) joint fluid white blood cell count, (c) % changes of joint circumference, (d) % changes of the range of joint motion, (e) joint fluid interleukin-1-beta concentration of the Ad-, rAAV2-, or scAAV2-injected joints, and (f) lameness grade. *Significantly greater than all other groups (*P* < 0.04). The Ad-injected joints showed significantly greater inflammatory responses compared to the rAAV2/scAAV2-injected joints, although all parameters were returned within normal range in 4-5 weeks after the joint injection.

The Ad vectors induced greater articular inflammation compared to rAAV2 and scAAV2 vectors with significantly greater joint fluid cell counts (*P* < 0.03: Figure 3(b)), joint circumference (*P* < 0.04: Figure 3(c)), and reduced range of joint motion (*P* < 0.02: Figure 3(d)), joint fluid IL-1*β* concentration (*P* < 0.001: Figure 3(e)), and lameness grade (*P* < 0.04: Figure 3(f)), although all parameters were recovered to normal values within 4-5 weeks. For the rAAV2- or scAAV2-injected joints, inflammatory parameters were increased, but not significantly different than GBSS-injected joints, except a significantly greater joint fluid protein concentration at day 2 (Figure 3(a)). None of inflammatory parameters were significantly different between GBSS and rAAV2 or scAAV2 at low dose, and scAAV2 at high dose. None of joint fluid samples from Ad/AAV2-injected joints showed detectable concentration of IL-1Ra protein.

**Figure 4:**
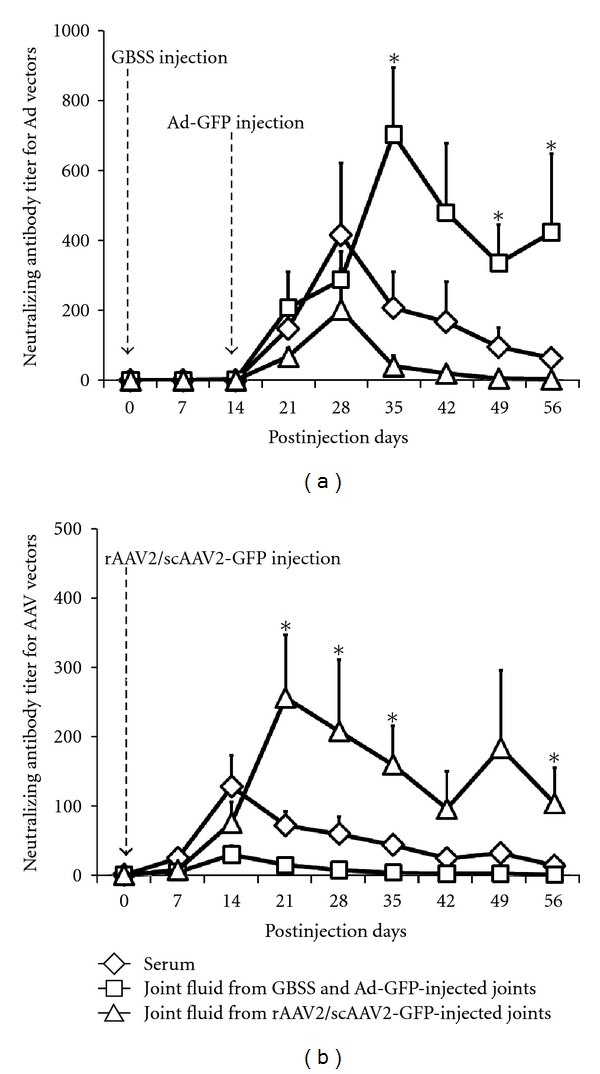
(a) The titer of neutralizing antibodies (NAb) against adenoviral (Ad), and (b) the titer of NAb against recombinant adeno-associated viral (rAAV2), or self-complementary AAV (scAAV2) vectors, in the serum, saline- (GBSS) and Ad-injected joint fluid, rAAV- or scAAV-injected joint fluid, or contralateral uninjected joint fluid. *Significantly greater than serum Ad/AAV2 NAb titers. There were no significant differences in NAb titers between types of AAV2 vectors (i.e., rAAV2 versus scAAV2). Both Ad and AAV2 vectors induce the sustained NAb production in the injected joint fluid and the transient NAb production in the serum and contralateral joint fluid.

The NAb titers against Ad- or AAV2 were significantly greater in the Ad- or AAV2-injected joint fluid compared to the serum or uninjected contralateral joint fluid (*P* < 0.03: Figures 4(a) and 4(b)), and injected synovial fluid titers remained high (>100) to the end of study period. The NAb titers for both serum and uninjected contralateral joint fluid against Ad or AAV2 peaked earlier at 2 weeks after intra-articular administration compared to injected synovial fluid (Figures 4(a) and 4(b)).

## 4. Discussion

Self-complementary AAV2 vectors showed promising and superior efficacy in vitro. Equine chondrocytes and synovial cells showed greater, more rapid, and sustained gene transduction by scAAV2 vectors compared to rAAV2 (Figures 2(a) and 2(b)). As the result, even 100 times lower dosage of scAAV2-GFP vectors (1 × 10^4^ DRP/cell) was able to induce the approximately equal % transduction to rAAV2-GFP vectors (1 × 10^6^ DRP/cell) in equine chondrocytes (Figure 2(b)). Bypassing rate-limiting second-strand DNA synthesis is expected to be largely beneficial to transfect the certain types of cells with low DNA replication; therefore, use of scAAV2 vector may be greatly advantageous for an intra-articular administration due to the low cell division rate of chondrocytes [14]. Because scAAV vectors do not require the second-strand DNA synthesis step, more rapid and superior gene expression of scAAV over rAAV vectors is expected in human chondrocytes and synovial cells as well. Our study also showed that, for the first time, there were no significant differences in the inflammation and immune response between the intra-articular rAAV2 and scAAV2 vectors (Figures 3(a) to 3(f) and Figure 4(b)). This is an expected result because the rAAV2 and scAAV2 vectors used in this study were the same serotype and contained the same capsid proteins, where the innate and adaptive immune responses are primarily initiated by recognition of the capsid proteins [20, 21]. An advantage of scAAV2 vectors may be detected in humans that have NAb titer against wild-type AAV2 (80% of human population) in that lower vector dosage may be effective and incite less immune reaction. Previous studies showed that, while the preexisting immunity by host exposure of the wild-type AAV2 is a significant limiting factor for rAAV2 gene transfer, the humoral immunity to the AAV2 capsid could be prevented by lowering the AAV2 particles administered [22]. 

Our study showed that AAV2 vectors may be a safer vector of choice for intra-articular gene administration, as they induced significantly less inflammatory response in the joints than Ad vector (Figure 3). The inflammation shown in the Ad-injected joints was expected and was transient. The signs of swelling, pain, and increased joint fluid protein and cell counts were significantly greater in Ad vectors compared to rAAV2 or scAAV2 vectors. Importantly, all parameters were returned to normal values within 4-5 weeks (Figure 3). Our results support that the adverse effects associated with an intra-articular Ad vector injection is temporary and in this study was clinically acceptable. The inflammation upon repeat injection was not studied in our report, but may be greater than first injection based on an amnestic immune response to the Ad vector.

To the authors' knowledge, this is the first study to report that intra-articularly administered Ad and AAV2 vectors induced antibody production in both serum and joint fluid. Previously, an intra-articular AAV2 injection has shown to increase serum antibody in human clinical trial [23]. The NAb production in the joint fluid may inhibit the repetitive injections of the gene delivery vectors in the same joint. A previous study reported that, after intra-articular IL-1Ra gene transduction by scAAV2 vectors, the reinjection of the vectors could not generate detectable levels of IL-1Ra expression [3]. In this regard, modification of the vectors might be necessary to prevent the immune responses that might interfere with successful repeat gene therapy application, including the capsid epitope alterations to decrease immunogenicity of the vector [24], cross-packaging of genomes into different AAV2 capsid serotype [25], or the application of transient immunosuppression at the time of the second vector administration [26]. Interestingly, the NAb titers in the Ad- or AAV2-injected joints were greater than serum titer and remained high (>100 NAb titer) until the end of the study period, even after the serum Ad or AAV2 titers were faded (Figures 4(a) and 4(b)). This may indicate the persistence of vectors in the injected joints.

Another important finding in our study is that, for both Ad and AAV2 vectors, the NAb titers against Ad/AAV2 vectors were increased in the synovial fluid from not only the Ad/AAV2-injected joint but also the contralateral joint where Ad/AAV2 vectors were not administered (Figures 4(a) and 4(b)). The increment and peak of the NAb titer in the contralateral joints corresponded to the serum NAb titer suggesting an infiltration of Ad antibodies from systemic circulation into the contralateral joint cavity. This may imply that treating one joint with viral vector may limit an efficacy of repeated application of intra-articular gene therapy in both the previously treated joints and the distant joints, at least for 6–8 weeks after the initial joint injection. A similar phenomenon has been noticed with intraocular administration of AAV2 vectors resulting in systemic antibody production and blocked transgene expression upon readministration in the contralateral eye [27].

Our study supported the safety of intra-articular gene therapy using AAV vectors in human joints. In this study, the direct intra-articular administration of relatively high dosage of rAAV/scAAV vectors (1 × 10^13^ DRP/joint) did not cause any detrimental inflammatory responses in equine metacarpo/tarsophalangeal joints with an average normal synovial fluid volume of 4.4 mL [28]. Osteoarthritis and rheumatoid arthritis commonly occur in human knee or elbow joints with an average normal synovial fluid volume of 4.5 mL [29]. In the literature, successful IL-1Ra or marker gene transductions of the articular cartilage and synovium were reported by the intra-articular administration of 4.7 × 10^11^ particles of rAAV in the mice knee joints [9], 5 × 10^11^ particles of scAAV in the rabbit knee joints [3], and 1.5 × 10^12^ particles of rAAV in the rabbit knee joints [11]. No significant inflammatory effects were evident in those reports, whereas the capacities of these rodent joints (0.5 mL) were estimated to be equal to human metacarpal-phalangeal joints that are a frequent site of rheumatoid arthritis [3].

## 5. Conclusion

Our results confirmed our hypothesis that scAAV2 vectors would show accelerated and greater gene transduction in vitro compared to rAAV2 and induce less inflammation and immune response in vivo compared to Ad vectors. Specifically, our results confirmed that (1) the scAAV2 vectors can induce superior gene transduction than rAAV2 in articular cells, and (2) both rAAV2 and scAAV2 were showed to be safer vectors for intra-articular administration by inducing significantly less joint inflammation than Ad vectors. Increased NAb titer by both AAV2 and Ad vectors may reduce effectiveness on repeat joint injection.

## List of Abbreviations

AAV2: Serotype 2 adeno-associated virus
Ad: Adenovirus
ANOVA: Analysis of variance
CMV: Cytomegalovirus
DMEM: Dulbecco's modified eagle medium
DRP: DNase-resistant particle
ELISA: Enzyme-linked immunosorbent assays
GBSS: Gey's balanced salt solution
GFP: Green fluorescent protein
IL-1Ra: Interleukin-1 receptor antagonist
NAb: Neutralizing antibody
rAAV2: Serotype 2 recombinant adeno-associated virus
scAAV2: Serotype 2 self-complementary adeno-associated virus.
